# Modulating Applied Task Performance *via* Transcranial Electrical Stimulation

**DOI:** 10.3389/fnhum.2019.00140

**Published:** 2019-04-30

**Authors:** Tad T. Brunyé, Erika K. Hussey, Eduardo B. Fontes, Nathan Ward

**Affiliations:** ^1^Center for Applied Brain and Cognitive Sciences, School of Engineering, Tufts University, Medford, MA, United States; ^2^U.S. Army Combat Capabilities Development Command, Soldier Center (CCDC-SC), Natick, MA, United States; ^3^Department of Psychology, Tufts University, Medford, MA, United States; ^4^NEUROEX-Research Group in Physical Activity, Cognition and Behavior, Health Science Center, Department of Physical Education, Federal University of Rio Grande do Norte, Natal, Brazil

**Keywords:** neuroergonomics, transcranial electrical stimulation, vigilance, multitasking, driving, navigation, virtual environments

## Abstract

Basic and applied research are increasingly adopting transcranial electrical stimulation (tES) for modulating perceptual, cognitive, affective, and motor processes. Industry and defense applications of tES hold potential for accelerating training and knowledge acquisition and sustaining work-related performance in the face of fatigue, workload, and stress. This mini-review article describes the promises and perils of tES, and reviews research testing its influence on two broad applied areas: sustaining and dividing attention, and operating in virtual environments. Also included is a discussion of challenges related to viable mechanistic explanations for tES effectiveness, attempts at replication and consideration of null results, and the potential importance of individual differences in predicting tES influences on human performance. Finally, future research directions are proposed to address these challenges and help develop a fuller understanding of tES viability for enhancing real-world performance.

## Introduction

Transcranial electrical stimulation (tES) involves administering low intensity electrical current to superficial cortical regions by way of two or more electrodes mounted on the surface of the scalp (Nitsche et al., 2008; Silva et al., 2008; Woods et al., 2016). Electrical current propagates through the scalp, skull, and dura mater and into cortical tissue; experiments with animals and modeling efforts demonstrate that tES can produce subthreshold depolarization of cortical pyramidal and glial cells (Ruohonen and Karhu, 2012; Molaee-Ardekani et al., 2013; Rahman et al., 2013). This is only one of many putative explanations for tES effects on human performance, the mechanisms of which remain somewhat elusive (Bestmann et al., 2015). It is generally accepted that tES modulates neuronal and neurotransmitter activity, underlying its effects on perceptual, cognitive, affective and motor processes seen in clinical, rehabilitation, educational, and recreational contexts. There are several methods for administering tES in the laboratory, with multiple methodological parameters demonstrated to independently and/or interactively influence the specificity, directionality, robustness and reliability of tES effects on human performance (Paulus, 2011; Reed and Cohen Kadosh, 2018; Yavari et al., 2018). This is a large and complex parameter space that deserves considerable attention from the scientific communities and is an important hurdle to overcome before tES can be applied reliably, selectively, and safely in real-world contexts.

This complexity has been compounded by a recent commercialization of tES for applications outside of the laboratory (Wurzman et al., 2016). It is important to note that most consumer-grade tES devices are not regulated by the United States Food and Drug Administration (FDA), are not considered low risk (FDA, 2016), and should be viewed with skepticism. Technological advances have, however, opened the door for neuroergonomics research to pursue tES research outside of the laboratory, which will benefit from the theories, tools, and techniques developed in the lab. Understanding the circumstances under which tES reliably alters human performance will help define and prioritize the real-world contexts and tasks that may prove suitable for tES application. The present review considers two broad areas that may prove fruitful for translational tES research: sustaining and dividing attention, and operating in virtual environments (Table 1).

**Table 1:** Overview of primary brain regions implicated in applied tasks, and existing research examining transcranial electrical stimulation (tES) effects on each applied task category.

Applied task	Brain regions implicated	Existing tES research
Sustained attention	Lateral prefrontal cortices Parietal lobes Temporoparietal region	McIntire et al. (2014), Nelson et al. (2014), Mauri et al. (2015), Kasten et al. (2016) and Loffler et al. (2018)
Threat detection and identification	Lateral PFC Anterior cingulate cortex Amygdala	Clark et al. (2012) and McKinley et al. (2013)
Divided attention, multitasking	Dorsolateral PFC Intraparietal sulcus Posterior lateral PFC Cerebellum	Filmer et al. (2013a,b), Nelson et al. (2016), Hsu et al. (2017) and Hsu et al. (2018)
Navigation and wayfinding	Medial and right inferior parietal cortex Posterior cingulate cortex Left PFC Medial temporal region Parahippocampal gyrus Hippocampus Retrosplenial complex	Brunyé et al. (2014, 2018a) and Hampstead et al. (2014)
Vehicle driving	Dorsolateral PFC Pre-supplementary motor area Superior parietal cortex Lateral occipital cortex Cerebellum	Beeli et al. (2008a,b) and Sakai et al. (2014)

## tES and Applied Tasks

Most tES research concerned with human performance focuses on modulating cognitive control processes by stimulating the dorsolateral prefrontal cortex (DLPFC; Brunoni and Vanderhasselt, 2014). The DLPFC supports a range of processes involved in controlling top-down control of complex voluntary actions, including solving complex tasks, inhibiting habitual responding, and correcting errors (MacDonald et al., 2000; Koechlin and Summerfield, 2007). Traditionally, DLPFC is targeted using bipolar electrode montages (i.e., one anode and one cathode). Complementing this work, researchers have begun to use multi-electrode montages arranged to target additional brain regions guided by finite element modeling, such as the hippocampus (Nikolin et al., 2015; Brunyé et al., 2018a), resting state motor network (Fischer et al., 2017), temporoparietal junction (Slaby et al., 2015), and inferior frontal cortex (Hussey et al., 2015; Hogeveen et al., 2016). Beyond adjusting the spatial properties of montages, recent work has also examined the temporal parameters of tES, including modifying dose (i.e., multiple days; longer administration times; Iyer et al., 2005; Reis et al., 2009; Hill et al., 2016), delivery time (i.e., online vs. offline stimulation; Pirulli et al., 2013), and frequency or phase properties (i.e., transcranial random noise stimulation, or tRNS; transcranial alternating current stimulation, or tACS; Filmer et al., 2014; Santarnecchi et al., 2015). This methodological variability is pervasive across basic and applied domains.

### Sustaining and Dividing Attention

Sustained attention, or vigilance, involves maintaining alertness and focus to goal-related but infrequent stimuli over extended periods of time (Davies and Parasuraman, 1982). Example work-related tasks include airport luggage screening, air traffic control, military checkpoints and image analysis, industrial quality control, driving, and many medical tasks such as histology screening (Warm et al., 2008). In these cases, operators are trained to maintain attentiveness to ensure the detection of rare but valuable targets. Studies have shown that vigilance declines as the time on task increases, as reflected by a decrease in target detections and increase in reaction times (Mackworth, 1948; Helton et al., 2007; Warm and Parasuraman, 2009). Functional magnetic resonance imaging (fMRI) and positron emission tomography (PET) have consistently revealed that sustaining attention recruits a network of brain regions including lateral prefrontal cortices, parietal lobes, and the temporoparietal region (Pardo et al., 1991; Coull et al., 1998; Breckel et al., 2011), making vigilance tasks an ideal target for tES.

Several studies have examined the link between prefrontal tES and vigilance task performance. First, Nelson et al. (2014) examined whether tDCS targeting the left DLPFC with anodal stimulation (relative to cathodal stimulation over the same region) would influence performance on a simulated air traffic control task. Anodal stimulation caused an increase in hit rates and decrease in false alarm rates relative to the cathodal and sham conditions, and this effect persisted throughout the 40-min task. Thus, there was some evidence that anodal tDCS targeting the left DLPFC can reduce some of the typical vigilance decrements seen in hit and false alarm rates, but not reaction times. In a second study, McIntire et al. (2014) examined whether anodal tDCS over the left DLPFC (with an extra-cephalic cathode) would mitigate sleep-deprivation induced vigilance decrements. They found that tDCS (vs. sham) induced sustained performance on two vigilance tasks (a psychomotor vigilance task and the Mackworth clock task), in the form of accuracy and response times. Using tACS, studies are equivocal: one showed no reliable effect of alpha-band tACS targeting posterior brain areas on visual vigilance performance (Kasten et al., 2016), and another showed a reduced vigilance decrement with gamma-band tACS over the visual cortex (Loffler et al., 2018). Using tRNS, one study showed that tRNS reduced reaction times during sustained vigilance on a continuous performance task (Mauri et al., 2015).

Some vigilance tasks involve highly specialized knowledge, such as with medical imaging, airport luggage screening, and monitoring radar systems for emergent threats (Lesgold et al., 1988; Patel et al., 2005; Ericsson et al., 2006). Some research has assessed whether tES may hold value for accelerating the acquisition of knowledge and skills required to successfully perform threat detection and identification tasks (Parasuraman and McKinley, 2014). Clark et al. (2012) administered anodal tDCS to the right inferior frontal cortex or right parietal cortex, and assessed the rate and amount of learning in a concealed object learning task. They found that both tDCS montages produced robust and reliable increases in accuracy, reflecting accelerated learning of the vigilance and threat detection task. A second study found similar results with anodal tDCS targeting the ventrolateral PFC, with increased performance on a learning task involving threat detection (object recognition; McKinley et al., 2013). Thus, there is some evidence that anodal tDCS targeting the PFC can reduce the typical vigilance decrements seen with extended time on task, and accelerate the learning of critical cues that are important for detecting threats during vigilance tasks.

Just as sustaining attention underlies a wide range of outcomes, dividing attention, or multitasking is a common demand imposed by several real-world tasks. Decades of research have demonstrated considerable costs to accuracy and response times when operators attempt to multitask, and these decrements are typically attributed to central interference imposed by competing tasks and responses (sometimes referred to as a central bottleneck; Pashler, 1994; Marois and Ivanoff, 2005). The posterior lateral PFC (pLPFC) has been identified as playing a critical role in the stimulus-response mappings that underlie effective dual-tasking components of multitasking (Jiang and Kanwisher, 2003; Dux et al., 2006, 2009). A few studies have examined whether tES targeting the left pLPFC would modulate multitasking performance, and results are equivocal. One study examined whether anodal or cathodal stimulation of the left pLPFC would modulate reaction times on simultaneous auditory and visual tasks (Filmer et al., 2013a). Cathodal tDCS reduced typical multitasking costs relative to anodal or sham stimulation, suggesting that cathodal stimulation may reduce neural noise and increase the signal to noise ratio in the pLPFC (Dockery et al., 2009; Miniussi et al., 2013). In a second study, the same research group demonstrated that anodal and cathodal tDCS targeting the left pLPFC reduced the typical performance advantages seen with multitasking training (Filmer et al., 2013b). Thus, there is evidence that cathodal pLPFC stimulation may improve multitasking performance, but that anodal or cathodal stimulation of the same region may interfere with multitasking training effectiveness. In both cases, the authors point to a critical causal role of this brain region for stimulus-response mapping, though the discrepant findings remain unresolved (Nikolin et al., 2019).

In a final study, Nelson et al. (2016) targeted the left DLPFC with anodal tDCS and found increased multitasking throughput capacity relative to sham stimulation. It is possible that a relatively domain-general increase in attentional control *via* DLPFC stimulation can induce enhancements on multitasking tasks, without specifically targeting the pLPFC (Strobach and Antonenko, 2017). This possibility is supported by studies demonstrating enhanced dual-tasking performance with tDCS targeting the inferior frontal (Strobach et al., 2015) and dorsolateral prefrontal cortices (Zhou et al., 2014). There is also evidence that tACS targeting theta oscillations in the PFC can induce multitasking improvements (Hsu et al., 2017, 2018). No research has directly examined the effects of tRNS on multitasking performance, though some suggest limited utility of tRNS for altering aspects of executive function reliant on the PFC (Mulquiney et al., 2011).

### Operating in Virtual Environments

Virtual reality tools are becoming increasingly integrated into applied psychology paradigms to create immersive, lifelike scenarios that mimic the demands encountered outside of the lab. While combining neurostimulation and virtual reality holds potential for several applied domains (Teo et al., 2016), two specific examples are navigation and driving.

Navigating between waypoints in large-scale environments relies on a collection of cognitive processes, including spatial attention, perception, mental rotation, visualization, and working memory (Byrne et al., 2007). This diversity of processing demands is matched by a highly distributed network of brain regions (Vogt et al., 1992; Brotchie et al., 1995; Xu and Chun, 2006; Burgess, 2008; Harvey et al., 2012; Wiener et al., 2016; Boccia et al., 2017), each of which presents targeting opportunities for tES (Brunyé, 2018). In one study, researchers targeted the right medial temporal lobe with a multi-electrode tDCS montage and demonstrated no main effect of anodal stimulation on the ability to efficiently navigate a virtual environment (Brunyé et al., 2014); they did, however, find some evidence that it benefitted individuals with relatively low spatial sense of direction. In a second virtual navigation study, researchers used finite element modeling to inform electrode positions targeting the right hippocampus and parahippocampus with anodal tDCS, and found no significant influence on a virtual navigation task involving spatial encoding and memory retrieval (Brunyé et al., 2018a). Continuing research may benefit from leveraging functional connectivity between cortical and subcortical regions, targeting cortical targets to indirectly modulate brain activity in medial brain regions (Hampstead et al., 2014; Brunyé, 2018), and possibly extending research to tACS and tRNS.

Like spatial navigation, vehicle driving engages a multitude of perceptual and cognitive processes, resulting in effectively processing driving-related information and translating that into effective and safe control strategies. One brain region frequently implicated in this task is the right and left DLPFC: studies demonstrate activity in the right DLPFC in a vehicle-following task requiring the maintenance of a specific lead distance, left DLPFC activity in a rural driving simulation, and right DLPFC activity when attending to traffic rules during driving (Spiers and Maguire, 2007; Just et al., 2008; Uchiyama et al., 2012). Still others find no evidence of DLPFC activity during simulated or actual vehicle driving tasks (Calhoun et al., 2002; Horikawa et al., 2005; Jeong et al., 2006).

To our knowledge, only three published studies to date have examined tES influences on driver behavior, all using tDCS (no tACS or tRNS). In the first, Beeli et al. (2008a) administered anodal or cathodal tDCS over the left or right DLPFC during simulated driving, and found that both anodal stimulation conditions caused a reduction of risky driving behavior (increased following distance, reduced speed). Another study replicated these findings and demonstrated that speed violations and revolutions per minute were also reduced during a simulated driving scenario with anodal tDCS over left or right DLPFC relative to sham (Beeli et al., 2008b). Finally, Sakai et al. (2014) administered anodal or cathodal stimulation over the right or left DLPFC, and found that right anodal improved following distances and lane-keeping performance relative to sham or left anodal stimulation. Thus, there is some evidence that variations of DLPFC stimulation can enhance certain safety-related aspects of driving.

## tES Challenges for Application

We point to three primary challenges in considering tES for application to applied contexts and tasks. First, safe and effective tES administration relies on a highly complex and underspecified parameter space (Soekadar et al., 2016). These include variation in targeted brain regions, electrode positioning and sizing, stimulation intensity, timing and duration, and the polarity (anodal, cathodal) of tDCS and frequency of tACS. Furthermore, challenges associated with the state-dependence of the cortex and differential responsiveness to tES (Dockery et al., 2009; Bikson et al., 2013; Antal et al., 2014) may prove especially important for real-world applications. Specifically, the complexity of endogenous neural activations during real-world tasks may modulate tES effects on behavior in unknown ways. These parameters are complicated by the complexity of current propagation through tissue and varied neuronal morphology, the non-linear excitability gradients of tES, varied device quality and reliability, and the potential for participants to detect differences between active and sham conditions. These challenges warrant caution among practitioners who seek to apply tES in contexts outside of the laboratory where they may realize reduced control over these factors. This is especially relevant given the heightened interest in extending applied cognitive tasks to immersive, ambulatory virtual environments, particularly in rehabilitation contexts (Rothwell, 2012; Viana et al., 2014; Massetti et al., 2017).

Second, recent research has suggested that individual differences in brain morphology, knowledge, skills and abilities play a role in predicting tES impacts. As noted previously, spatial skills predicted whether tDCS influenced navigation performance (Brunyé et al., 2014); it could be the case that those with high spatial skills have relatively optimized neural network dynamics during complex spatial tasks, and tES can interfere with those relatively refined activity patterns. Similar influences of individual differences in predicting tES influences have been found in several recent studies: high creative potential is linked to increased breadth of semantic associations with left frontopolar tDCS (Brunyé et al., 2015), working memory capacity and educational level are linked to increased neural activity and cognitive function with left DLPFC tDCS (Berryhill and Jones, 2012; Jones et al., 2015), and trait mathematics anxiety is linked to whether left DLPFC tDCS improves arithmetic task performance (Sarkar et al., 2014). Continuing research will likely find additional predictive value in trait-based measures, and the field will continue to benefit from neuroimaging in at least three ways: individualized targeting of brain regions (Bikson et al., 2012), closed-loop stimulation triggering using structural and functional brain imaging (McKendrick et al., 2015), and improved mechanistic understandings of tES effects at the level of neurons, neural networks, and behavior (Filmer et al., 2014; Soekadar et al., 2016).

Third, at least partially due to the complex parameter space of tES and individual variability in the robustness and directionality of behavioral responses, it is important to consider studies demonstrating null and negative effects of tES. Recent meta-analyses are equivocal in determining whether tES induce reliable effects on perceptual and cognitive processes in healthy adults (Jacobson et al., 2012; Chhatbar and Feng, 2015; Horvath et al., 2015a,b). Furthermore, some studies have found unexpected negative results of tDCS targeting the DLPFC, suggesting that tES is not a one-size-fits-all solution for all individuals, contexts, and tasks (Zwissler et al., 2014; Crivelli and Balconi, 2017; Brunyé et al., 2018b). Moving forward, we argue for the use of more rigorous and reproducible methods in tES research, especially when it comes to less standardized applied tasks and outcome measures. This may include establishing required sample sizes, procedures, predicted effect sizes, and analysis approaches in advance (e.g., through preregistered reports). Furthermore, while tDCS is increasingly used in relatively applied contexts, research using tACS and tRNS in applied domains is largely limited to areas such as motor learning (Nitsche et al., 2003) and procedural skill acquisition (Tecchio et al., 2010).

## Conclusion

tES may prove valuable for modulating applied task performance, though research in this area warrants careful consideration of several individual-, context-, and task-related factors that may predict the robustness and directionality of tES effects. Whereas most applied research with tES has administered tDCS, tACS and tRNS have also shown potential to modulate cortical activity and behavior. Even in highly applied and dynamic tasks, such as navigation and driving, tES appears to carry some performance benefits. This is compelling because as tES is slowly incorporated into highly complex real-world environments and tasks, there is potential that its robustness and reliability may diminish relative to results found in controlled laboratory and simulation environments. Continuing research will benefit from transitioning tES out of the laboratory and simulation environment and examining such a possibility.

## Author Contributions

TB conceived the review and prepared the manuscript, with critical feedback and revisions from EH, EF, and NW.

## Conflict of Interest Statement

The authors declare that the research was conducted in the absence of any commercial or financial relationships that could be construed as a potential conflict of interest.
